# Modeled small airways lung deposition of two fixed-dose triple therapy combinations assessed with *in silico* functional respiratory imaging

**DOI:** 10.1186/s12931-023-02534-y

**Published:** 2023-09-23

**Authors:** Omar Usmani, Grace Li, Jan De Backer, Hosein Sadafi, Libo Wu, Jonathan Marshall

**Affiliations:** 1 Imperial College London and Royal Brompton Hospital, London, UK; 2 AstraZeneca, South San Francisco, CA USA; 3 FLUIDDA Inc, New York, NY USA; 4 FLUIDDA NV, Kontich, Belgium; 5 AstraZeneca, Durham, NC USA; 6 AstraZeneca, Academy House, 136 Hills Road, Cambridge, CB2 8PA UK

**Keywords:** Budesonide/glycopyrronium/formoterol fumarate dihydrate (BGF), Chronic obstructive pulmonary disease (COPD), Fluticasone furoate/umeclidinium/vilanterol (FF/UM/VI), *In silico* functional respiratory imaging (FRI), Lung deposition, Inhaled corticosteroids (ICS), Triple therapy

## Abstract

**Background:**

Small airways disease plays a key role in the pathogenesis of chronic obstructive pulmonary disease (COPD) and is a major cause of obstruction; therefore, it is a critical pharmacotherapy target. This study evaluated lung deposition of two inhaled corticosteroid (ICS)/long-acting β_2_-agonist/long-acting muscarinic antagonist single-inhaler triple therapies using *in silico* functional respiratory imaging (FRI). Deposition was assessed using real-world inhalation profiles simulating everyday use where optimal inhalation may be compromised.

**Methods:**

Three-dimensional airway models were produced from 20 patients with moderate-to-very severe COPD. Total, central, and regional small airways deposition as a percentage of delivered dose of budesonide/glycopyrronium/formoterol fumarate dihydrate (BGF) 160/7.2/5 µg per actuation and fluticasone furoate/umeclidinium/vilanterol (FF/UM/VI) 100/62.5/25 µg were evaluated using *in silico* FRI based on in vitro aerodynamic particle size distributions of each device. Simulations were performed using multiple inhalation profiles of varying durations and flow rates representing patterns suited for a pressurized metered-dose inhaler or dry-powder inhaler (four for BGF, two for FF/UM/VI, with one common profile). For the common profile, deposition for BGF versus FF/UM/VI was compared post-hoc using paired t-tests.

**Results:**

Across inhalation profiles, mean total lung deposition was consistently higher with BGF (47.0–54.1%) versus FF/UM/VI (20.8–22.7%) and for each treatment component, with greater deposition for BGF also seen in the central large airways. Mean regional small airways deposition was also greater across inhalation profiles with BGF (16.9–23.6%) versus FF/UM/VI (6.8–8.7%) and for each treatment component. For the common profile, total, central, and regional small airways deposition were significantly greater for BGF versus FF/UM/VI (nominal p < 0.001), overall and for treatment components; notably, regional small airways deposition of the ICS components was approximately five-fold greater with budesonide versus fluticasone furoate (16.1% vs. 3.3%).

**Conclusions:**

BGF was associated with greater total, central, and small airways deposition for all components versus FF/UM/VI. Importantly, using an identical inhalation profile, there was an approximately five-fold difference in small airways deposition for the ICS components, with only a small percentage of the ICS from FF/UM/VI reaching the small airways. Further research is needed to understand if the enhanced delivery of BGF translates to clinical benefits.

**Graphical Abstract:**

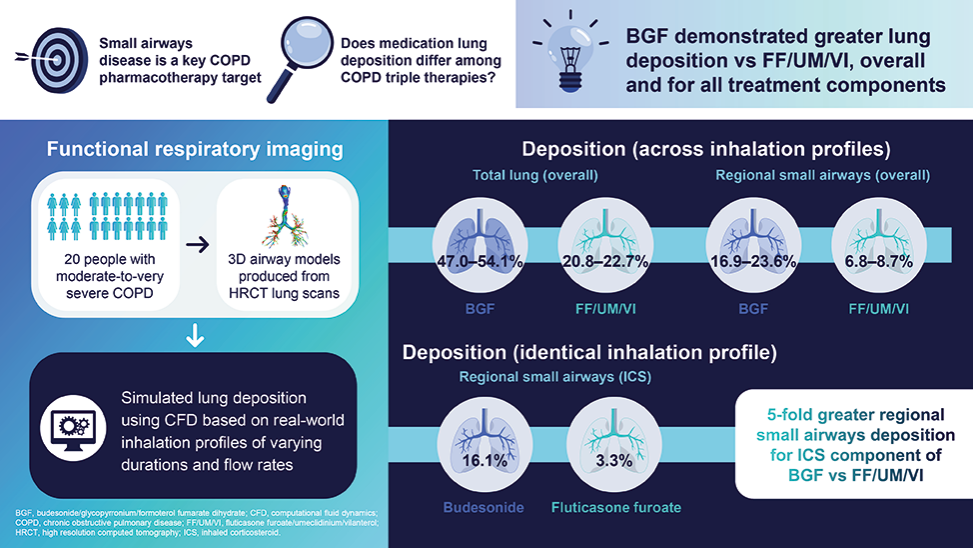

**Supplementary Information:**

The online version contains supplementary material available at 10.1186/s12931-023-02534-y.

## Introduction

Small airways disease plays a key role in chronic obstructive pulmonary disease (COPD) and is a major cause of airway obstruction; therefore, it is a critical target of pharmacotherapy [1, 2]. Multiple single-inhaler inhaled corticosteroid/long-acting muscarinic antagonist/long-acting β_2_-agonist (ICS/LAMA/LABA) therapies are approved for COPD maintenance treatment. In the United States and the European Union, approved treatments for COPD include budesonide/glycopyrronium/formoterol fumarate dihydrate (BGF) 320/14.4/10 µg via pressurized metered dose inhaler (pMDI) [3, 4] and fluticasone furoate/umeclidinium/vilanterol (FF/UM/VI) 100/62.5/25 µg via dry powder inhaler (DPI) [5, 6]. Beclometasone dipropionate/formoterol fumarate dihydrate/glycopyrronium bromide (BDP/F/GB) 87/5/9 µg via pMDI is also approved in the European Union [7].

In large Phase III clinical trials, all three of these single-inhaler ICS/LAMA/LABA triple therapies have demonstrated improved efficacy versus ICS/LABA dual therapy [8–11] and LAMA/LABA dual therapy [8, 9, 12, 13], including reductions in COPD exacerbation rates. Furthermore, BGF and FF/UM/VI have demonstrated reductions in mortality compared with LAMA/LABA dual therapy [14, 15].

It has been shown that different inhaled treatment formulations achieve different lung deposition patterns [16], which could be associated with differential clinical effects in patients with COPD [17]. Additionally, different inhalation profiles have been shown to affect both aerodynamic particle size distribution (APSD), particularly with DPIs [18], and lung deposition profiles [19]. Suboptimal inspiratory flow and inhalation technique errors have also been associated with higher COPD-related healthcare utilization and costs in patients with COPD using DPI maintenance therapy [20].

The aim of this study was to evaluate total, central, and regional small airways deposition of BGF and FF/UM/VI using *in silico* functional respiratory imaging (FRI), a technology that uses computational fluid dynamics (CFD) applied to 3D computational models developed from high resolution computed tomography (HRCT) lung scans of patients [21]. Importantly, the study focused on assessing inhaled lung deposition with different patient inhalation profiles that reflect the recommended techniques for pMDIs and DPIs and everyday real-world use [22], where optimal inhalation technique may be compromised.

## Methods

### Study design

*In silico* FRI is a technique that combines HRCT scans with CFD and provides quantitative insight on several endpoints in the respiratory domain. The *in silico* FRI methodology utilized in this study assesses the transport of inhaled compounds in respiratory systems by applying CFD to patient-specific airway geometries.

*In silico* FRI methodology has been previously described and validated against combined single photon emission CT and CT (SPECT/CT) [21], with strong consistency demonstrated versus results with scintigraphy for multiple disease populations and treatments (Additional File 1: Table S1). In brief, the technique comprises four components: (1) patient HRCT scanning to produce three-dimensional (3D) airway models; (2) determination of inhaler characteristics; (3) setting the inhalation profiles; (4) *in silico* lung deposition modeling using CFD simulations.

#### 3D airway models

To produce the 3D airway models, HRCT lung scans were obtained from 20 patients with moderate-to-very severe COPD. There was no active patient recruitment for this study; patient HRCT scans were obtained retrospectively from the FLUIDDA database. For this study, HRCT scans were selected based on the type of patients encountered in clinical trials (based on forced expiratory volume in 1 s [FEV_1_]) and the type of patients using these treatments in the real world. The HRCT scans were taken at total lung capacity and functional residual capacity.

The 3D airway models included the extrathoracic region (mouth and upper airways) and the intrathoracic airways. Regions of interest were not defined. The whole lung, including lobes and airways, was extracted. The segmentation and 3D model operations were performed using commercially available validated software packages (Mimics 20.0 and 3-Matic 12.0, Materialise nv, Belgium). The models were converted to tetrahedral 3D volume meshes using TGrid 14.0 and computationally solved in Fluent 14.0 (Ansys Inc, Canonsburg, PA, USA). The HRCT scans and lower airway segmentations were previously developed, but the 3D airways models are unique to this study.

#### Inhaler characteristics

The in vitro aerosol performance of each treatment was characterized by a next-generation impactor using the method described in United States Pharmacopoeia (USP) chapter ⟨601⟩ [23]. Based on unpublished data from AstraZeneca, the plume characteristics for the BGF pMDI were fixed as follows: angle 27.4°; velocity (distance from edge of mouthpiece) 16.2 m/s at 25 mm, 8.2 m/s at 75 mm, and 7.1 m/s at 100 mm; injection duration 0.18 s. The values of velocity at three distances from the nozzle were used to extrapolate the velocity magnitude at the nozzle based on the approach previously described by Talaat et al. [24]. The injection duration for the FF/UM/VI DPI was fixed at 0.3 s based on author experience; this time value is supported by the literature [25], although the current model assumed that drug was released at a constant rate over time.

For BGF pMDI, two actuations of 160/7.2/5 µg (i.e. one dose of 320/14.4/10 µg) were tested and at two different flow rates of 30 l/min and 60 l/min. For FF/UM/VI DPI, one actuation of 100/62.5/25 µg (i.e. one dose) was tested and at two different flow rates of 30 l/min and 76 l/min. The 76 l/min flow rate for FF/UM/VI DPI was selected to generate a pressure drop of 4 kPa following USP chapter ⟨601⟩ [23].

#### Inhalation profiles

The inhalation profiles had varying duration and flow rate (peak and mean) to reflect different patterns of real-world use and abilities of patients with obstructive lung disease, some being more optimal than others depending on the device used.

**Fig. 1:**
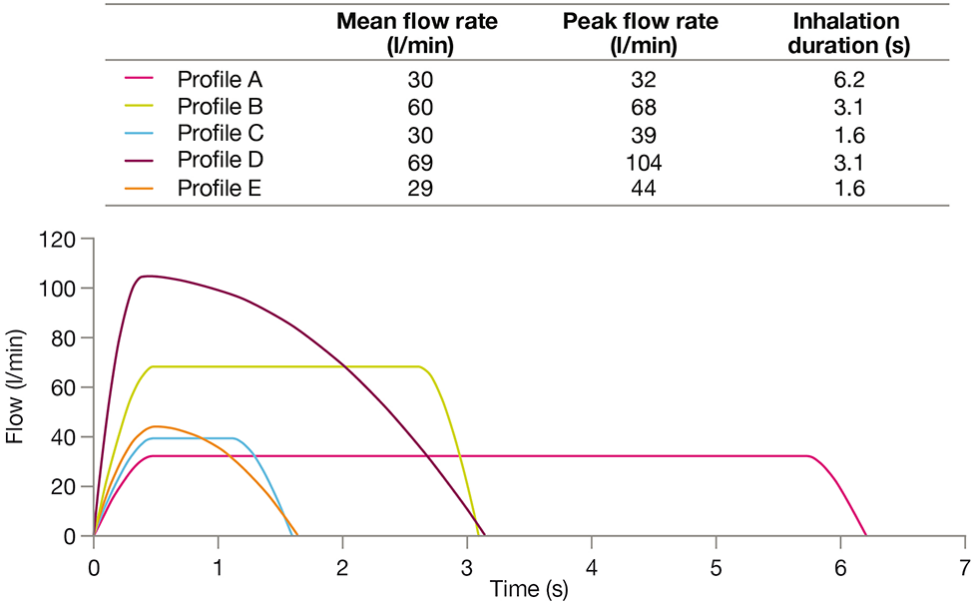
Inhalation profiles

Some profiles would be considered to be more suited for pMDI (smooth and consistent) and others more suited for DPI (rapid acceleration and slow decline) (Fig. 1). For the inhalation profiles suited for DPI, the inhalation times and volumes were based on those of patients with COPD found in the literature [26]. The inhalation profiles suited for pMDI were derived from the European Respiratory Society (ERS) and International Society for Aerosols in Medicine (ISAM) task force report guidelines on how to breathe optimally through a pMDI [27]. BGF was tested with profiles A–D and FF/UM/VI was tested with profiles D–E (Fig. 1): profile A – mean flow rate 30 l/min, inhalation duration 6.2 s; profile B – mean flow rate 60 l/min, inhalation duration 3.1 s; profile C – mean flow rate 30 l/min, inhalation duration 1.6 s; profile D – mean flow rate 69 l/min, inhalation duration 3.1 s; profile E – mean flow rate 29 l/min, inhalation duration 1.6 s. Profile D was chosen for comparison of the treatments under the same conditions, as it was expected to be the most optimal profile for DPI and the least optimal for pMDI.

#### Lung deposition modeling

The patient HRCT scans were used to generate models of each inhaler, with each inhaler’s geometry reverse-engineered from the HRCT into a 3D computer-aided design model. The position of the lips on the mouthpiece of the pMDI or DPI inhaler was determined to ensure that the inhaler would be inserted at the correct depth. The inhaler was then virtually coupled with the upper airway models to ensure that air would flow through the device towards the opening of the mouth and not up to the hard palate or down to the tongue.

In the simulations, the flow motion was governed by the conservation of mass, momentum, and energy equations. The air flow was coupled with the discrete phase (aerosols) using source terms in the conservation equations. To model the turbulence effects, the Large Eddy Simulation turbulence model was used [28].

To represent real-life conditions, *in silico* FRI technology applies patient-specific boundary conditions in the CFD simulations. As such, the lobe volumes and airway geometries of each real patient were extracted from their inspiratory and expiratory HRCT scans. The ventilated air from each lobe was then determined and applied as the correct flow rate from the airways. Since these quantities were not modeled but measured in vivo, physical phenomena including the peripheral resistance and regional compliance were automatically involved in the process.

The APSD data were fed into the CFD simulations to simulate particle motion and predict airway deposition for the various inhalation profiles. Lung deposition was evaluated in the intrathoracic airways (total lung), divided into the central airways and the regional small airways. The central airways were defined as those up to approximately ninth-generation bronchioles (approximately 2 mm in diameter; captured by HRCT scan). The regional small airways, also known as the peripheral airways [2], were defined as those beyond the limits of HRCT scanning, i.e. after approximately the ninth-generation bronchioles. Particles not deposited in the extrathoracic regions or in the central airways were considered to be deposited in the regional small airways as it was assumed that no particles were exhaled with a recommended breath-hold.

Data are reported as the percentage of the delivered dose of BGF (one actuation of 160/7.2/5 µg) or FF/UM/VI (one actuation of 100/62.5/25 µg), overall and for the individual ICS, LAMA, and LABA treatment components. Although the approved dose of BGF is two actuations of 160/7.2/5 µg (i.e. one dose is 320/14.4/10 µg), lung deposition is expressed as a percentage of delivered dose, and each actuation would be expected to provide almost identical results; thus, it was only necessary to model one actuation of BGF to estimate lung deposition.

### Statistical methods

All statistical analyses were conducted using R version 3.2.5 or higher (The R Foundation for Statistical Computing, Vienna, Austria). Lung deposition data are presented in boxplots in which: the data labels are mean ± standard deviation (SD); the extremes of the box are the upper and lower quartiles; the horizontal line within the box is the median; the whiskers extend to the most extreme data points no more than 1.5 times the interquartile range; individual data points beyond the whiskers are outliers beyond 1.5 times the interquartile range.

A post-hoc analysis was performed using paired t-tests to compare total lung deposition, regional small airways deposition and central lung deposition for BGF versus FF/UM/VI for each treatment component (ICS, LAMA and LABA), and for the overall average of the combination of components. Comparisons were performed using deposition data for the common inhalation profile (profile D: mean flow rate 69 l/min, peak flow rate 104 l/min, inhalation duration 3.1 s), producing mean difference (and 95% confidence intervals [CIs]) in lung deposition between treatments and nominal p-values for these differences.

## Results

### Participants

Six patients were female and 14 were male. As determined based on The Third National Health and Nutrition Examination Survey (NHANES III) reference equations, 16 patients had moderate-severe COPD and four patients had very severe COPD. Mean (SD) age was 64.65 (8.27) years and mean (SD) height was 169.4 (9.41) cm. Mean (SD) pre-bronchodilator FEV_1_ was 1.27 (0.59) l, mean (SD) pre-bronchodilator FEV_1_% predicted was 44.09 (14.2), and all patients had > 120% predicted functional residual capacity.

### Particle characteristics

**Fig. 2:**
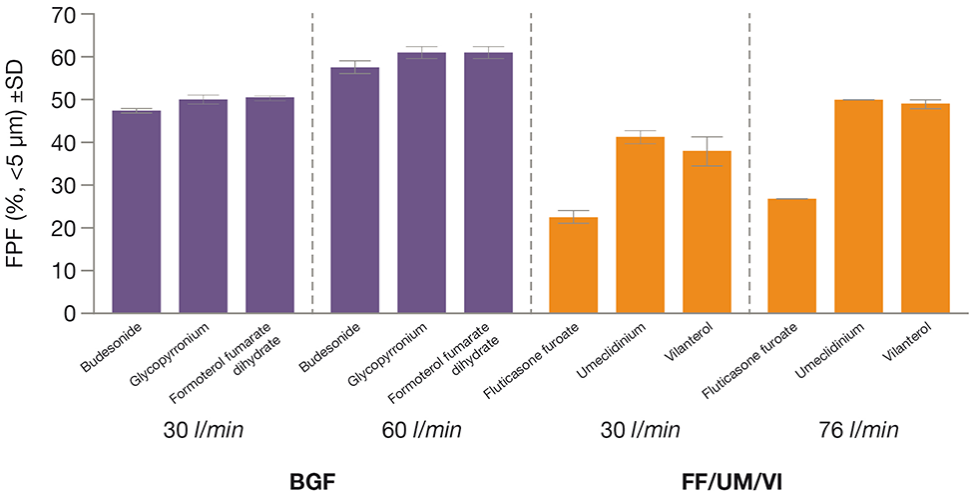
Fine particle fraction for BGF and FF/UM/VI at different flow rates BGF, budesonide/glycopyrronium/formoterol fumarate dihydrate; FF/UM/VI, fluticasone furoate/umeclidinium/vilanterol; FPF, fine particle fraction; SD, standard deviation

The in vitro aerosol performance of BGF and FF/UM/VI at different flow rates is presented in Table 1 and the fine particle fraction (FPF) at different flow rates is also depicted in Fig. 2.

**Table 1:** Particle characteristics for BGF and FF/UM/VI at different flow rates

	BGF					
Flow rate	30 l/min			60 l/min		
Treatment component	Budesonide	Glycopyrronium	Formoterol fumarate	Budesonide	Glycopyrronium	Formoterol fumarate
**Delivered dose (µg)**	159.0	7.2	4.6	148.9	6.8	4.3
**FPM (µg, < 5 μm)**	75.5	3.6	2.3	85.9	4.1	2.6
**FPF (%, < 5 μm)**	47	50	51	58	61	61
**MMAD (µm)**	3.5	3.3	3.3	3.4	3.1	3.1
**GSD**	1.6	1.6	1.7	1.7	1.7	1.8
	**FF/UM/VI**					
**Flow rate**	**30 l/min**			**76 l/min**		
**Treatment component**	**Fluticasone ** **furoate**	**Umeclidinium**	**Vilanterol**	**Fluticasone furoate**	**Umeclidinium**	**Vilanterol**
**Delivered dose (µg)**	89.4	54.7	23.5	88.7	55.1	23.0
**FPM (µg, < 5 μm)**	20.2	22.7	8.9	23.9	27.6	11.3
**FPF (%, < 5 μm)**	23	41	38	27	50	49
**MMAD (µm)**	4.4	3.0	2.4	3.8	2.7	1.9
**GSD**	2.1	2.0	2.3	2.1	2.0	2.3

BGF, budesonide/glycopyrronium/formoterol fumarate dihydrate; FF/UM/VI, fluticasone furoate/umeclidinium/vilanterol; FPF, fine particle fraction; FPM, fine particle mass; GSD, geometric standard deviation; MMAD, mass median aerodynamic diameter

For BGF, FPF was similar for the ICS, LAMA, and LABA treatment components, being 47–51% at 30 l/min and 58–61% at 60 l/min. The mass median aerodynamic diameter (MMAD) for BGF was also similar across treatment components, being 3.3–3.5 μm at 30 l/min and 3.1–3.4 μm at 60 l/min. The geometric standard deviation (GSD) for BGF was 1.6–1.7 at 30 l/min and 1.7–1.8 at 60 l/min across treatment components.

For FF/UM/VI, there were differences in particle characteristics across treatment components, with lower FPF and higher MMAD for the ICS component compared with the LAMA and LABA components. At 30 l/min, the FPF was 23% for the ICS component and 38–41% for the LAMA and LABA treatment components. At 76 l/min, the FPF was 27% for the ICS treatment component and 49–50% for the LAMA and LABA treatment components. The MMAD was 4.4 μm at 30 l/min and 3.8 μm at 76 l/min for the ICS component, 3.0 μm at 30 l/min and 2.7 μm at 76 l/min for the LAMA component, and 2.4 μm at 30 l/min and 1.9 μm at 76 l/min for the LABA component. The GSD across treatment components was 2.0–2.3 at 30 l/min and 2.0–2.3 at 76 l/min.

### Lung deposition

#### Lung deposition overall for BGF versus FF/UM/VI

**Fig. 3:**
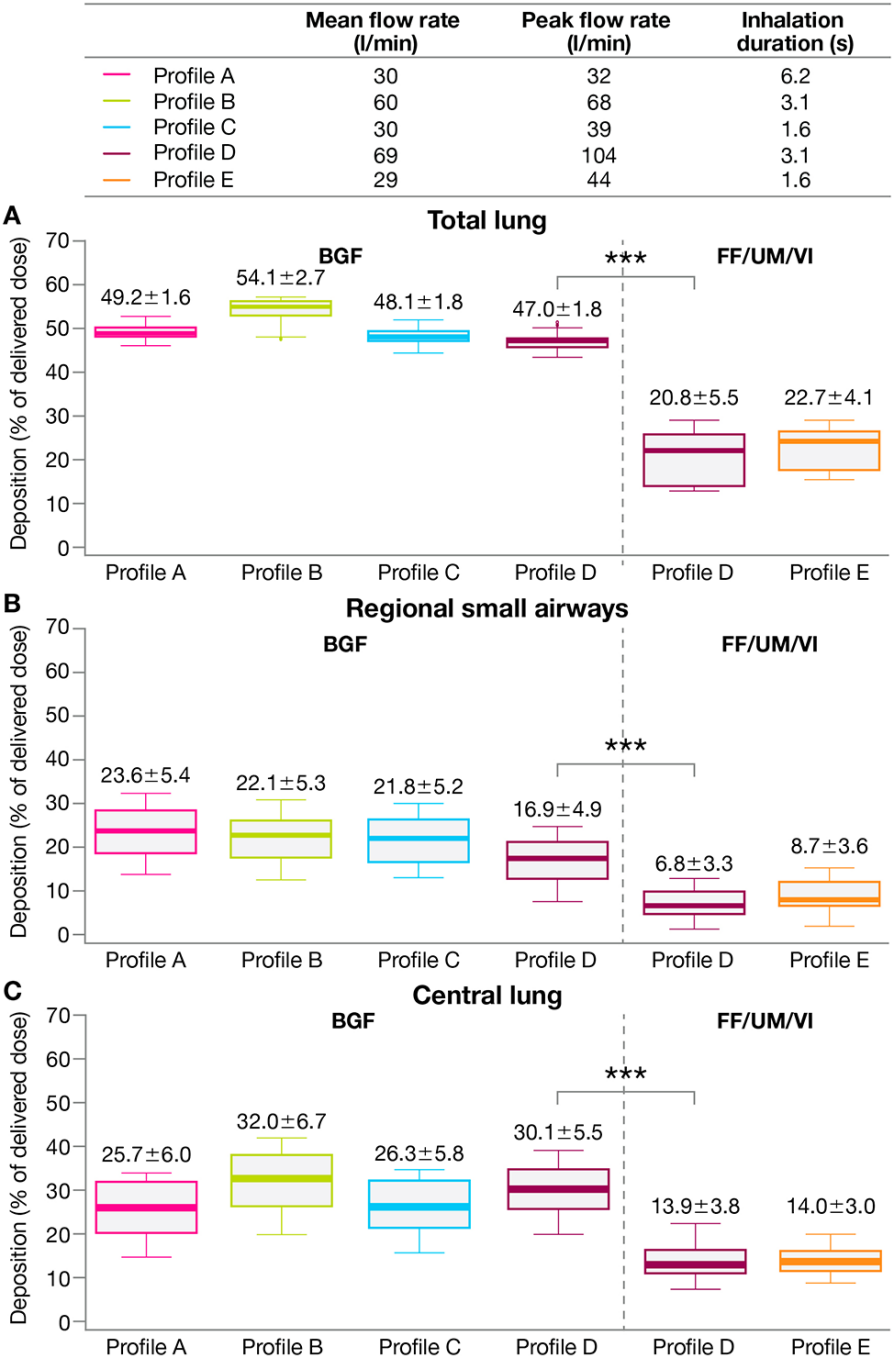
**A** Total lung, **B** regional small airways, and **C** central lung deposition (overall) by treatment ***Nominal p < 0.001 (paired t-test) for BGF versus FF/UM/VI based on mean difference (95% CI): total lung, 26.3 (24.9–27.7); regional small airways, 10.1 (9.2–11.0); central lung, 16.2 (15.0–17.5) Data labels: mean ± SD. Extremes of box: upper and lower quartiles. Horizontal line within box: median. Whiskers: extend to most extreme data points no more than 1.5 times the interquartile range. Individual data points beyond whiskers: outliers beyond 1.5 times the interquartile range BGF, budesonide/glycopyrronium/formoterol fumarate dihydrate; CI, confidence interval; FF/UM/VI, fluticasone furoate/umeclidinium/vilanterol; SD, standard deviation

**Fig. 4:**
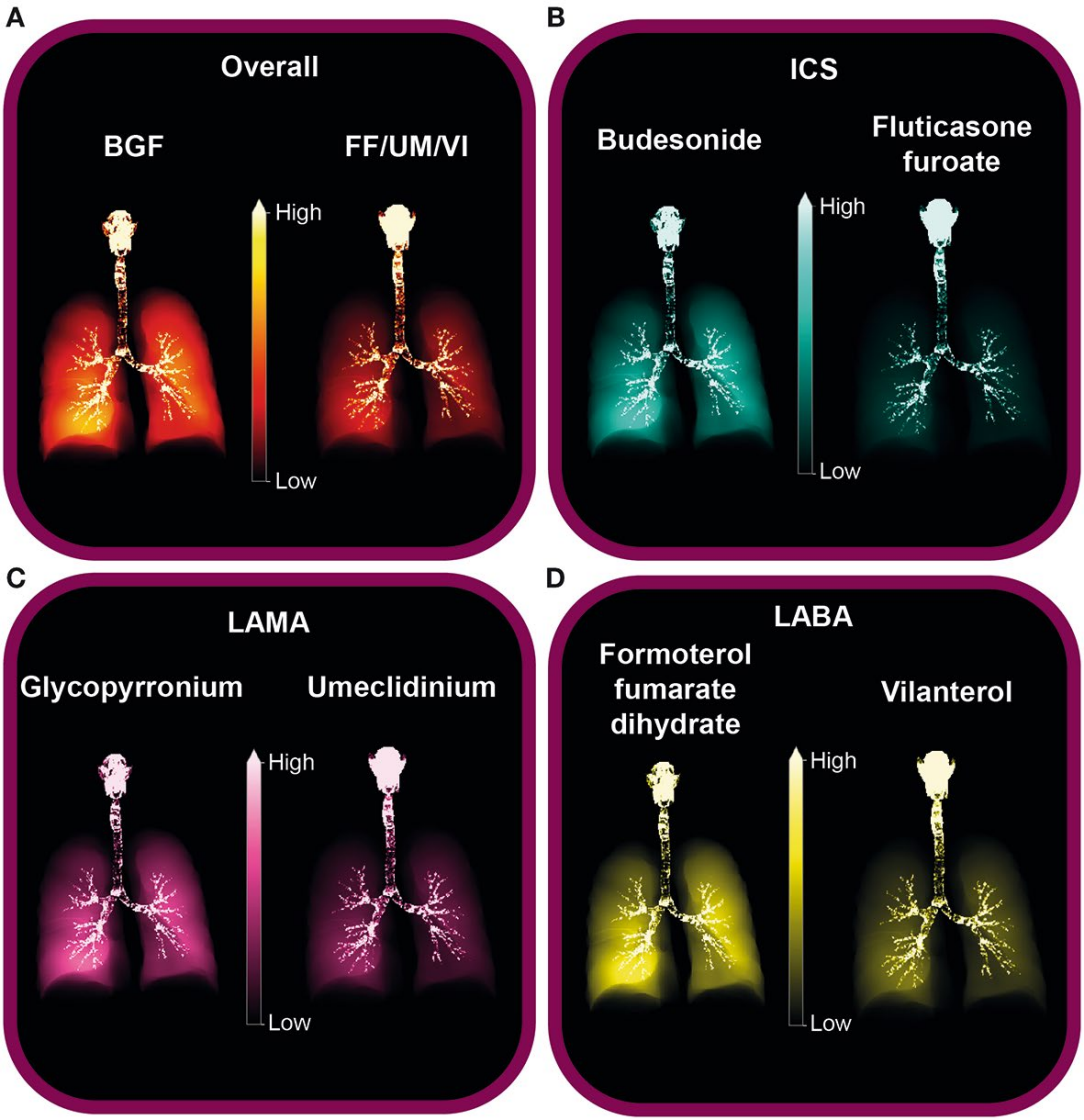
Visualization of deposition for profile D: **A** overall, **B** ICS, **C** LAMA, and **D** LABA Profile D inhalation profile: mean flow rate 69 l/min, peak flow rate 104 l/min, inhalation duration 3.1 s BGF, budesonide/glycopyrronium/formoterol fumarate dihydrate; FF/UM/VI, fluticasone furoate/umeclidinium/vilanterol; ICS, inhaled corticosteroid; LABA, long-acting β_2_-agonist; LAMA, long-acting muscarinic antagonist

Mean total lung deposition (Fig. 3A) and regional small airways deposition (Fig. 3B) as percentage of delivered dose were consistently higher with BGF versus FF/UM/VI across inhalation profiles. With the common inhalation profile (profile D: mean flow rate 69 l/min, peak flow rate 104 l/min, inhalation duration 3.1 s), mean total lung deposition as a percentage of delivered dose was significantly greater for BGF versus FF/UM/VI (47.0% vs. 20.8%; nominal p < 0.001) and mean regional small airways deposition as a percentage of delivered dose was also significantly greater for BGF versus FF/UM/VI (16.9% vs. 6.8%; nominal p < 0.001). Mean central lung deposition as a percentage of delivered dose was also consistently higher for BGF versus FF/UM/VI (Fig. 3C), including for the common inhalation profile (profile D; 30.1% for BGF vs. 13.9% for FF/UM/VI; nominal p < 0.001). The increased delivery of BGF versus FF/UM/VI with profile D can be seen in a representative visualization of the deposition patterns (Fig. 4A).

#### Lung deposition by treatment component (ICS, LAMA, and LABA)

**Fig. 5:**
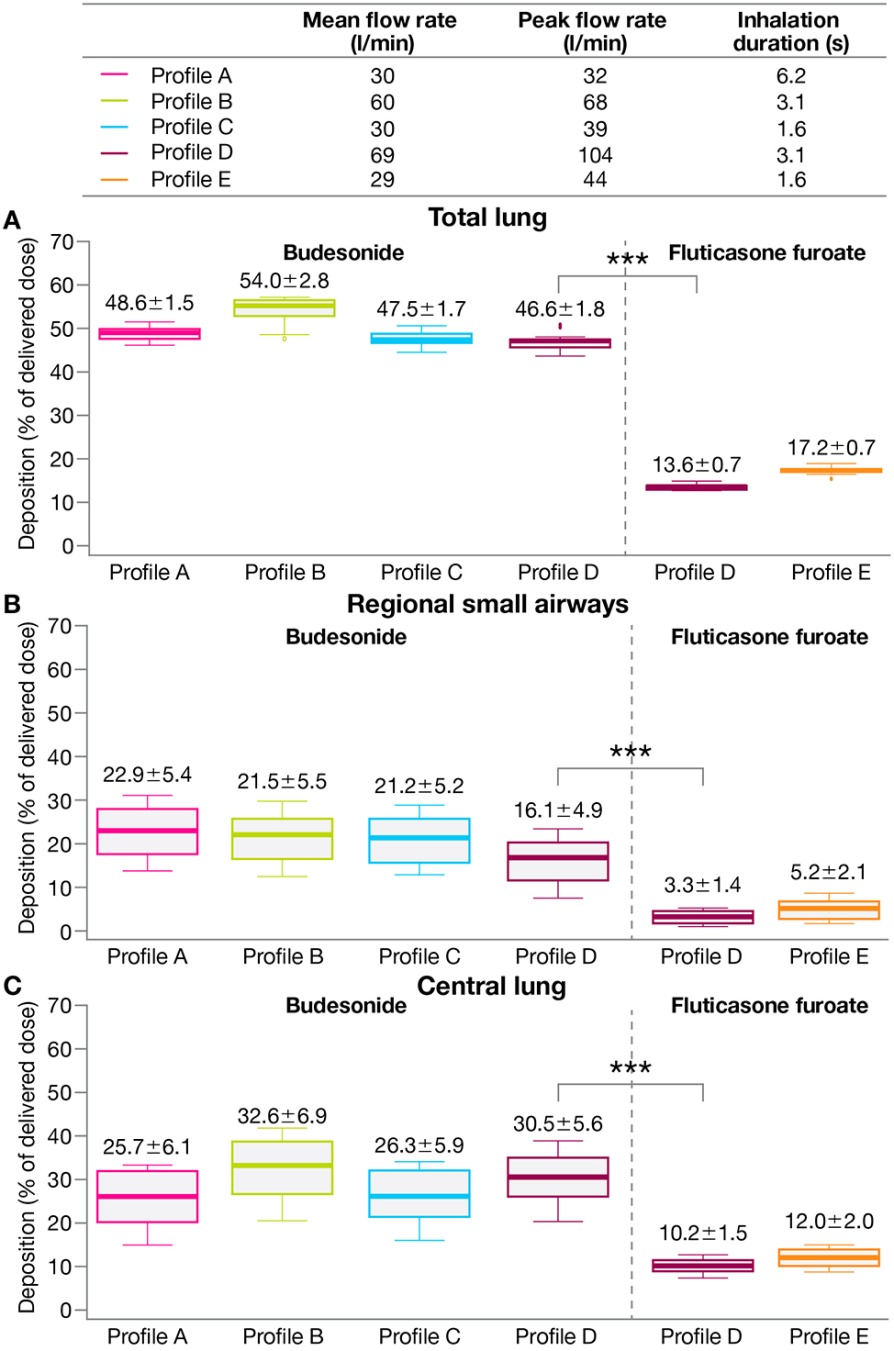
**A** Total lung, **B** regional small airways, and **C** central lung deposition for ICS components of each treatment ***Nominal p < 0.001 (paired t-test) for BGF versus FF/UM/VI based on mean difference (95% CI): total lung, 33.0 (32.2–33.9); regional small airways, 12.8 (11.1–14.5); central lung, 20.2 (18.2–22.2) Data labels: mean ± SD. Extremes of box: upper and lower quartiles. Horizontal line within box: median. Whiskers: extend to most extreme data points no more than 1.5 times the interquartile range. Individual data points beyond whiskers: outliers beyond 1.5 times the interquartile range BGF, budesonide/glycopyrronium/formoterol fumarate dihydrate; CI, confidence interval; FF/UM/VI, fluticasone furoate/umeclidinium/vilanterol; ICS, inhaled corticosteroid; SD, standard deviation

Mean total lung deposition (Fig. 5A), regional small airways deposition (Fig. 5B), and central lung deposition (Fig. 5C) as a percentage of delivered dose for each inhalation profile were consistently higher for the ICS component of BGF (budesonide) versus for the ICS component of FF/UM/VI (fluticasone furoate). The differences in lung deposition for budesonide versus fluticasone furoate with the common inhalation profile (profile D) were all statistically significant (nominal p < 0.001; Fig. 5A–C). Notably, regional small airways deposition of budesonide from BGF was approximately five-fold greater versus fluticasone furoate from FF/UM/VI (16.1% vs. 3.3%) with profile D (Fig. 5B).

Across inhalation profiles, mean total lung deposition, regional small airways deposition, and central lung deposition as a percentage of delivered dose were also higher for the LAMA components (glycopyrronium from BGF vs. umeclidinium from FF/UM/VI; Additional File 2: Fig. S1) and LABA components (formoterol fumarate dihydrate from BGF vs. vilanterol from FF/UM/VI; Additional File 3: Fig. S2) of BGF versus FF/UM/VI, and statistically significant differences (nominal p < 0.001) were consistently seen with profile D.

The increased delivery of each treatment component of BGF versus FF/UM/VI with profile D can be seen in a representative visualization of the deposition patterns (Fig. 4B–D).

## Discussion

In this *in silico* FRI modeling study, BGF demonstrated greater total lung deposition, central lung deposition, and small airways deposition compared with FF/UM/VI. These findings were consistently observed across different inhalation profiles of varying durations and flow rates representing recommended techniques for pMDIs and DPIs, as well as real-world usage where optimal inhalation may be compromised.

Correct inhaler technique is important for COPD treatment, as it ensures the appropriate delivery of medication [27], which is critical for medication efficacy [27]. As such, poor inhaler technique is associated with increased COPD-related healthcare utilization and costs compared with proper inhaler technique [20]. Per the ERS and ISAM task force report, multidisciplinary experts advise that patients should inhale slowly and deeply when using an pMDI, while for DPI usage, patients should inhale forcefully from the very beginning [27].

Interestingly, in this *in silico* FRI study, deposition (total lung, regional small airways, and central lung) of BGF and its components were not substantially affected by differences in inhalation profiles even though fast inhalation is reported to be one of the most common inhaler technique errors for pMDIs in patients with respiratory disease [29, 30]. In this study, inhalation profile D (mean flow rate 69 l/min, peak flow rate 104 l/min, inhalation duration 3.1 s) reflected a pattern of inhalation that would be expected to be more suited to DPI usage, as a rapid acceleration is needed to de-agglomerate powder into fine particles [27]. Importantly, the lung deposition findings for BGF with profile D demonstrate that BGF is tolerant of fast inhalation, mitigating the consequence of a rapid inhalation due to poor technique or poor inspiratory capacity. The BGF pMDI is formulated using co-suspension delivery technology whereby drug crystals are co-suspended with aerodynamic porous phospholipid particles that dissolve once they reach the airway surface, depositing the drug [17]. Previous studies have demonstrated that this inhaler provides optimal particle size and FPF, and effective delivery to the central and small airways, even in the presence of simulated patient handling errors [17, 31]. As such, the formulation technology may mitigate any impact on deposition caused by different inhalation profiles.

As mentioned previously, inhalation profile D reflects a pattern that would be recommended for DPI usage, with a high inspiratory flow rate, rapid acceleration, and slow decline. Profile D was tested for both BGF and FF/UM/VI and not only did the lung deposition findings demonstrate that BGF was tolerant of this inhalation profile, but also deposition was significantly greater (nominal p < 0.001) than that of FF/UM/VI.

Greater lung deposition was observed not only for BGF versus FF/UM/VI overall, but also for each treatment component (ICS, LAMA, and LABA). Notably, for profile D, there was an approximately five-fold difference in regional small airways deposition for the ICS components (16.1% for budesonide vs. 3.3% for fluticasone furoate; nominal p < 0.001). Both aerodynamic particle size and FPF are determinants of total and regional lung deposition [19]. Indeed, there may be a correlation between differences in the MMAD and FPF of the treatments and the difference in regional small airways deposition. For BGF, the MMAD and FPF were similar for the ICS, LAMA, and LABA treatment components; however, for FF/UM/VI, the MMAD was greater and the FPF was lower for the ICS component versus the LAMA and LABA components. Furthermore, across treatment components, the GSD was greater for FF/UM/VI than for BGF, indicating more variable particle size.

A key question is whether such differences in lung deposition could lead to clinical differences. Although both BGF and FF/UM/VI have demonstrated reductions in mortality versus dual LAMA/LABA therapy [14, 15], a recent publication using a matching-adjusted indirect comparison indicates reductions in mortality with BGF are greater than those with FF/UM/VI [32]. Possible explanations include pharmacokinetic and pharmacological differences, particularly of budesonide and fluticasone furoate, but the current study suggests that lung deposition, particularly of ICS, to the small airways could potentially contribute to such differences. This is supported by data from the ETHOS study, which showed that the 320 µg dose of budesonide in BGF resulted in a mortality benefit but the 160 µg dose did not [14] (i.e., more ICS to the lungs provided greater benefit). So, it is possible that mortality benefits of BGF versus FF/UM/VI are linked to the individual ICS, the dose, and the amount of lung deposition.

In addition to BGF and FF/UM/VI, BDP/F/GB is another COPD maintenance treatment approved in the European Union. Although this study did not evaluate lung deposition of BDP/F/GB, two similar *in silico* FRI studies have assessed lung deposition with BDP/F/GB in patients with COPD [33, 34]. In one study, total lung deposition of BDP/F/GB via pMDI was 31.0% (SD 5.7) with a real-world inhalation profile (mean flow rate 29 l/min) [34], which appears to be lower than the total lung deposition of BGF in the current study across all inhalation profiles assessed (47.0–54.1% [SD 1.6–2.7]). In the other study of BDP/F/GB, which again used real-world inhalation profiles (average flow rates ranged from 16 to 68 l/min), total lung deposition was 35.9% (SD 6.66) for the ICS component, 35.5% (SD 6.62) for the LAMA component, and 36.7% (SD 6.81) for the LABA component [33]. Thus, the total lung deposition for the BDP/F/GB treatment components in this previous study appears to be lower than for the treatment components of BGF in the current study, across all inhalation profiles assessed: ICS, 46.6–54.0% (SD 1.5–2.8); LAMA, 47.2–54.0 (SD 1.5–2.6); LABA, 47.3–54.3 (SD 1.5–2.7).

Lung deposition of BGF and BDP/F/GB has been evaluated separately for each treatment in gamma scintigraphy studies. The mean emitted dose of BGF deposited in the lungs was 32.1% in patients with moderate-to-very severe COPD, 35.2% in patients with moderate COPD, and 28.7% in patients with severe/very severe COPD [35]. In healthy male participants, the mean emitted dose of BGF deposited in the lungs ranged from 34.5 to 37.7% depending on length of breath hold [36]. Mean intrapulmonary deposition as percentage of metered dose of BDP/F/GB was 25.50% in patients with asthma and 22.74% in healthy volunteers [37]. Although this is an indirect comparison between studies, this would suggest that BGF has greater in vivo lung deposition than BDP/F/GB. However, it has been suggested that extrafine particles have greater ability to reach the small airways than larger particle formulations [1, 27]. It is therefore interesting that, comparing the findings for a similar breath-hold in healthy participants, the standardized central/peripheral ratio was 1.79 for BGF [36] and 1.80 for BDP/F/GB [37], demonstrating that fine (BGF) and extrafine (BDP/F/GB) particle formulations delivered via pMDI can target both central and peripheral airways. Further research is needed under like-for-like conditions to better understand the relative depositions of these two formulations.

The findings of this study must be interpreted in light of some limitations. A key limitation is that computational modeling is associated with some degree of uncertainty. It is not possible to fully replicate real-life settings, for example physiological factors including lung surface features and airway humidity [19, 38], and the complex process of aerosolization [19, 39]. However, CT scanning and modeling is increasingly being used to assess lung deposition [40]. *In silico* FRI has demonstrated consistency with SPECT/CT under similar conditions in patients with asthma [21] and consistency with scintigraphy in different populations [41–43], including patients with COPD; thus, *in silico* FRI has become an accepted estimate of lung deposition. Additionally, due to the limitations of the technology and resolution of the HRCT scans, the technique does not allow for the determination of the distribution of deposition within the small airways, but rather small airways distribution is treated as a single compartment. The technique also did not allow for prediction of any exhaled drug; it was assumed that no particles were exhaled with a recommended breath-hold. However, this assumption should have little impact on the findings, as a previous scintigraphy study of BGF deposition demonstrated that, even with a short breath-hold (3 s), a low percentage (mean 0.4%) of the emitted dose of BGF was detected in the exhalation filter [36]. Furthermore, whilst this study evaluated multiple flow rates and inhalation durations that reflect real-world inhaler usage, there are multiple inhaler technique errors that could impact delivery of drugs to the lungs [44], and it is not possible to model all aspects in a single study. A further limitation of this study is that the statistical comparisons of lung deposition for BGF versus FF/UMVI with profile D were post-hoc and p-values are nominal.

Key strengths of this study include that deposition was assessed using a range of real-world patient inhalation profiles simulating everyday use where optimal inhalation may be compromised. Furthermore, the *in silico* FRI technique allows for comparative studies and precise control of variables, including inhalation profiles, so that different inhalers are compared under like-for-like conditions and in the ‘same lungs’. Although the technique is based on computer modeling, it offers some advantages compared with traditional techniques for evaluating lung deposition (for example, scintigraphy), by making studies shorter and faster, and thus more cost-effective, as well as reducing, or even obviating, the need to expose patients to radiation.

## Conclusions

In this *in silico* FRI study, BGF was associated with greater total lung, central lung, and regional small airways deposition in patients with moderate-to-very severe COPD for all triple therapy components versus FF/UM/VI. Importantly, when predicting lung deposition using an identical inhalation profile, there was an approximately five-fold difference in regional small airways deposition for the ICS components (16.1% for budesonide in BGF vs. 3.3% for fluticasone furoate in FF/UM/VI; nominal p < 0.001), with only a small percentage of the ICS from FF/UM/VI reaching the small airways. Further research is needed to understand if the enhanced delivery of BGF translates to clinical benefits, such as greater reductions in mortality.

### Electronic supplementary material

Below is the link to the electronic supplementary material.

**Additional File 1: Table S1** Consistency of *in silico* FRI compared with scintigraphy

**Additional File 2: Fig. S1 A** Total lung, **B** regional small airways, and **C** central lung deposition for LAMA components of each treatment

**Additional File 3: Fig. S2 A** Total lung, **B** regional small airways, and **C** central lung deposition for LABA components of each treatment

## Data Availability

The datasets generated during and/or analyzed during the current study are available from the corresponding author on reasonable request.

## List of abbreviations

3D: Three-dimensional
APSD: Aerodynamic particle size distribution
BDP: Beclometasone dipropionate/formoterol fumarate/glycopyrronium bromide
BGF: Budesonide/glycopyrronium/formoterol fumarate dihydrate
CFD: Computational fluid dynamics
COPD: Chronic obstructive pulmonary disease
CT: Computed tomography
DPI: Dry powder inhaler
ERS: European Respiratory Society
FEV_1_: Forced expiratory volume in 1 s
FF/UM/VI: Fluticasone furoate/umeclidinium/vilanterol
FPF: Fine particle fraction
FPM: Fine particle mass
FRI: Functional respiratory imaging
GSD: Geometric standard deviation
HRCT: High resolution computed tomography
ICS: Inhaled corticosteroid
ISAM: International Society for Aerosols in Medicine
LABA: Long-acting β_2_-agonist
LAMA: Long-acting muscarinic antagonist
MMAD: Mass median aerodynamic diameter
pMDI: Pressurized metered-dose inhaler
USP: United States Pharmacopoeia
