# Supplementary material for: Modeled small airways lung deposition of two fixed-dose triple therapy combinations assessed with in silico functional respiratory imaging
Source: Respir Res. 2023 Sep 23;24:226. doi: 10.1186/s12931-023-02534-y (PMC10517457; doi:10.1186/s12931-023-02534-y)
Supplement: Supplementary file 1 — Additional File 1: Table S1 Consistency of in silico FRI compared with scintigraphy [file 12931_2023_2534_MOESM1_ESM.docx]

## Additional File 1: Table S1 Consistency of *in silico* FRI compared with scintigraphy

| **Treatment** | **Population** | ***In silico* FRI, % lung deposition^a^ [citation]** | **Scintigraphy, % lung deposition^a^ [citation]** |
| --- | --- | --- | --- |
| Beclomethasone dipropionate/formoterol fumarate via pMDI | COPD | 28 [1] | 33 [2] |
| Beclomethasone dipropionate/formoterol fumarate via pMDI | Asthma | 30 [3] | 31 [2] |
| Fluticasone propionate/formoterol fumarate via pMDI | Asthma | 42 [4] | 43 [5] |
| Budesonide via DPI | Asthma | 23 [6]^b^ | 22 [7]^c^ |

^a^All deposition values are reported as the study population average % of labelled dose

^b^Data for the budesonide component of budesonide/formoterol fumarate dual therapy; % of metered dose converted to % of labelled dose $(28.85\% \times\frac{160 \mu g}{200 \mu g}=23.08\%)$

^c^Data for budesonide monotherapy

COPD, chronic obstructive pulmonary disease; DPI, dry powder inhaler; FRI, functional respiratory imaging; pMDI, pressurized metered dose inhaler

1. Usmani OS, Mignot B, Kendall I, Maria R, Cocconi D, Georges G, et al: Predicting lung deposition of extrafine inhaled corticosteroid-containing fixed combinations in patients with chronic obstructive pulmonary disease using functional respiratory imaging: an in silico study. *J Aerosol Med Pulm Drug Deliv.* 2021;34(3):204–11.

2. De Backer W, Devolder A, Poli G, Acerbi D, Monno R, Herpich C, et al: Lung deposition of BDP/formoterol HFA pMDI in healthy volunteers, asthmatic, and COPD patients. *J Aerosol Med Pulm Drug Deliv.* 2010;23(3):137–48.

3. Vinchurkar S, Backer LD, Vos W, Holsbeke CV, Backer JD, Backer WD: A case series on lung deposition analysis of inhaled medication using functional imaging based computational fluid dynamics in asthmatic patients: effect of upper airway morphology and comparison with in vivo data. *Inhal Toxicol.* 2012;24(2):81–8.

4. Van Holsbeke C, De Backer J, Vos W, Marshall J: Use of functional respiratory imaging to characterize the effect of inhalation profile and particle size on lung deposition of inhaled corticosteroid/long-acting β_2_-agonists delivered via a pressurized metered-dose inhaler. *Ther Adv Respir Dis.* 2018;12:1753466618760948.

5. Kappeler D, Sommerer K, Kietzig C, Huber B, Woodward J, Lomax M, et al: Pulmonary deposition of fluticasone propionate/formoterol in healthy volunteers, asthmatics and COPD patients with a novel breath-triggered inhaler. *Respir Med.* 2018;138:107–14.

6. Iwanaga T, Kozuka T, Nakanishi J, Yamada K, Nishiyama O, Sano H, et al: Aerosol deposition of inhaled corticosteroids/long-acting β_2_-agonists in the peripheral airways of patients with asthma using functional respiratory imaging, a novel imaging technology. *Pulmonary Therapy.* 2017;3(1):219–31.

7. Hirst PH, Bacon RE, Pitcairn GR, Silvasti M, Newman SP: A comparison of the lung deposition of budesonide from Easyhaler, Turbuhaler and pMDI plus spacer in asthmatic patients. *Respir Med.* 2001;95(9):720–7.
