# Supplementary material for: Modeled small airways lung deposition of two fixed-dose triple therapy combinations assessed with in silico functional respiratory imaging
Source: Respir Res. 2023 Sep 23;24:226. doi: 10.1186/s12931-023-02534-y (PMC10517457; doi:10.1186/s12931-023-02534-y)
Supplement: Supplementary file 2 — Additional File 2: Fig. S1 A Total lung, B regional small airways, and C central lung deposition for LAMA components of each treatment [file 12931_2023_2534_MOESM2_ESM.docx]

## Additional File 2: Fig. S1 A Total lung, B regional small airways, and C central lung deposition for LAMA components of each treatment


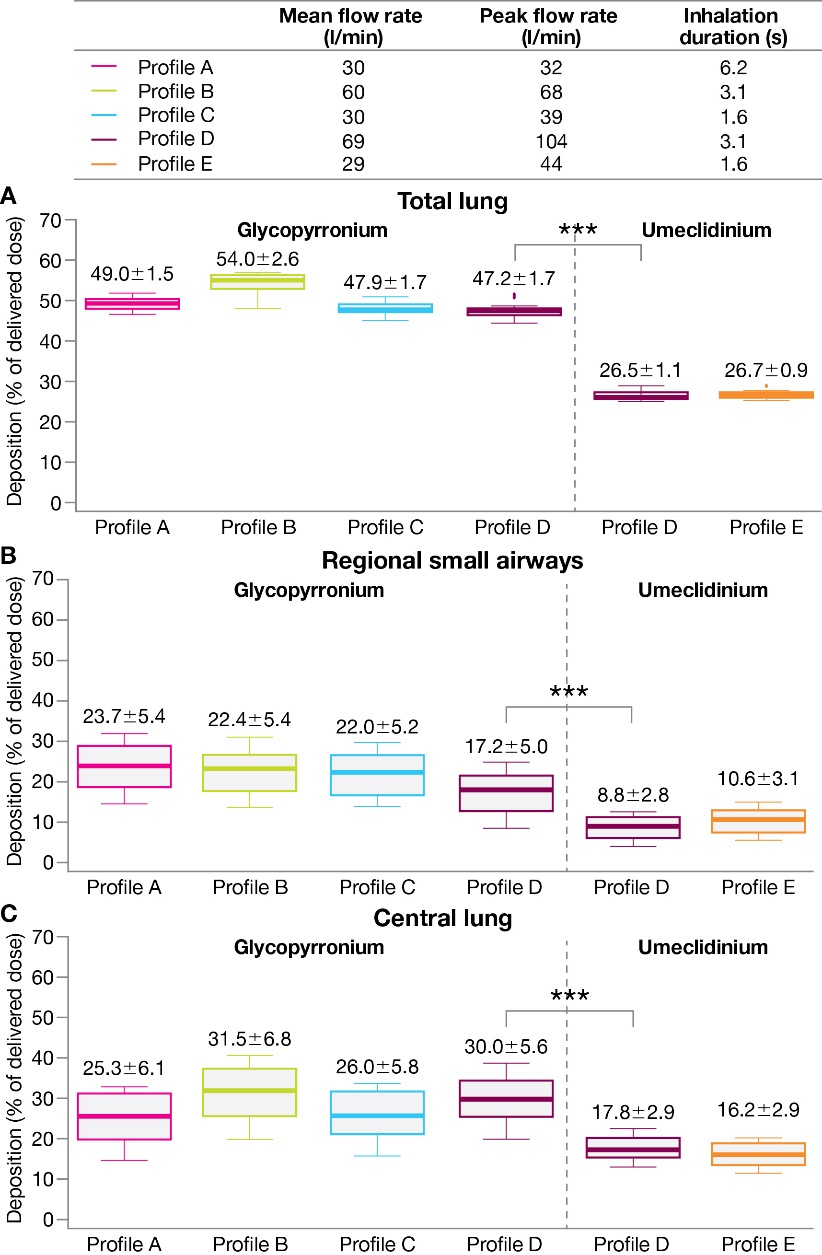


***Nominal p<0.001 (paired t-test) for BGF versus FF/UM/VI based on mean difference (95% CI): total lung, 20.7 (19.9–21.5); regional small airways, 8.4 (7.4–9.5); central lung, 12.3 (10.8–13.7)

Data labels: mean ±SD. Extremes of box: upper and lower quartiles. Horizontal line within box: median. Whiskers: extend to most extreme data points no more than 1.5 times the interquartile range. Individual data points beyond whiskers: outliers beyond 1.5 times the interquartile range

BGF, budesonide/glycopyrronium/formoterol fumarate dihydrate; CI, confidence interval; FF/UM/VI, fluticasone furoate/umeclidinium/vilanterol; LAMA, long-acting muscarinic antagonist; SD, standard deviation
